# Results from a Spanish national survey on the application of ultrasound in pulmonology services

**DOI:** 10.1186/s13089-021-00240-8

**Published:** 2021-08-24

**Authors:** Cristina Ramos-Hernández, Maribel Botana-Rial, Rosa Cordovilla-Pérez, Manuel Núñez-Delgado, Alberto Fernández-Villar

**Affiliations:** 1 Department of Pneumology, Hospital Alvaro Cunqueiro, Neumo Vigo I + I. Institute of Health Research Galicia South (IISGS), Xerencia de Xestión integrada de Vigo, C/Clara Campoamor 341, 36312 Vigo, Pontevedra Spain; 2 Department of Pneumology, University Hospital of Salamcanca, P.º de San Vicente, 182, 37007 Salamanca, Spain

**Keywords:** Ultrasound, LUS, Pulmonology, Survey

## Abstract

**Background:**

This was an observational, cross-sectional, and multicentre study carried out from October to December 2020, through a survey sent to Spanish Society of Pulmonology and Thoracic Surgery members in public hospitals with different levels of complexity. Our objective was to complete a national analysis of clinical practice, organisation, infrastructure, the services portfolio, teaching, and research activity related to ultrasound.

**Results:**

Data from 104 hospitals were analysed. Ultrasound was used in 56.7% of cases, both in the area of bronchopleural techniques and on conventional wards, with no differences between centres. Lung ultrasound (LUS) was performed more often in the procedures area in intermediate-complexity centres compared to high- and low-complexity centres (36% vs. 31% and 6.25%, respectively). More high-complexity centres had three or more ultrasound scanners than intermediate-complexity centres (38% vs. 16%); 43% of low-complexity centres shared their ultrasound equipment with other specialties. Fewer than 6% of centres did not have an ultrasound machine. LUS was most often used during the treatment of pleural effusion (91.3%), in the differential diagnosis of dyspnoea (51.9%), and to rule out iatrogenic pneumothorax (50.9%). Only 5.7% of the centres had a pulmonologist specialised in LUS. Finally, fewer than 35% of the hospitals were teaching centres and fewer than 18% participated in research projects.

**Conclusions:**

The use and availability of LUS has grown in pulmonology services, however, still relatively few pulmonologists are specialised in its use. Moreover, teaching and research activity in this field is scarce. Strategies are necessary to improve physicians’ skill at using LUS and to promote its use, with the ultimate goal of improving healthcare activity.

## Background

The use of ultrasound in pulmonology services is evident, not only because of the increase in its indications but also because of the number of specialists who have incorporated it into their daily clinical practice. Although, ultrasound initially started to be used as a guide during pleural procedures [1], over time it has proven useful in the assessment of the lung parenchyma, diaphragm, and chest wall, and it has became a basic first-line tool to establish a differential diagnosis in patients with dyspnoea [2, 3]. The use of ultrasound by pulmonologists has also extended to the evaluation of the lower limbs [4], vocal chords [5], and even for performing echocardiography [6] at the patient’s bedside to obtain data that may be useful in the diagnosis of pulmonary pathologies. Lung ultrasound (LUS) is a technique performed in bronchopleural procedures units and in intermediate respiratory care units, but its use in conventional hospital floor settings or in pneumology consultations is not as well established.

It is important to understand the degree to which ultrasound is used in the field of pulmonology at the national level to help researchers to detect potential difficulties in further developing this technique and to create recommendations or training programs that target the fields that need them the most, which helps physicians to achieve higher quality care. Thus, we carried out this study to evaluate the current situation in clinical settings and to address the issues set out above. Our main objective was to describe current clinical care practices regarding the use of ultrasound and to collect data related to its use in pulmonology services, organisational and infrastructural factors, the portfolio of services available, and ultrasound teaching and research activity.

## Methods

### Design

From October to December 2020, we carried out a cross-sectional, national level, multicentre study through a survey sent to every member of the Spanish Society of Pulmonology and Thoracic Surgery (SEPAR) working in hospitals with different levels of complexity. The latter factor was evaluated considering the criteria used in other national studies [7]. High-complexity centres were considered as those with a large technological endowment, more than 500 beds, and 160–300 medical intern residents (MIRs); intermediate-complexity centres were those with 200–500 beds and more than 50 MIRs; and low-complexity centres had fewer than 200 hospital beds and/or fewer than 50 MIRs.

Every pulmonologist and MIR in this specialty who were SEPAR members and who may be involved in the routine use of LUS (in hospital wards, consultations, intermediate respiratory care units, or bronchopleural procedures area) were sent the study survey. Privately managed centres and those located outside the national territory were excluded. When responses were obtained from several professionals from the same centre, the answers from the first participant were recorded.

Using a methodology similar to one our group has previously employed in a similar study [7], SEPAR members were contacted with the questionnaire by email and asked to complete it using an online platform. The survey included a total of 21 items, four of them designed to assess technological resources and the organisation and infrastructure available for LUS (Table 1), 12 items aimed to assess the portfolio of services available using this technique (Table 2), and five items were used to understand the centre’s involvement in teaching, training, and research related to ultrasound (Table 3).

**Table 1:** Organisational and infrastructural factors related to the use of ultrasound

Organisation and infrastructure	Total *N* = 104	High-complexity centre *N* = 58	Intermediate-complexity centre *N* = 30	Low-complexity centre *N* = 16
Availability of ultrasound scanners				
Two exclusive-use ultrasound scanners are available in the pneumology service	24/104 (23.0%)	17/58 (29.3%)	5/30 (16.6%)	2/16 (12.5%)
Three or more exclusive-use ultrasound scanners are available in the pneumology service	27/104 (25.9%)	22/58 (37.9%)^a^	5/30 (16.6%)	0/16 (0%)
An ultrasound system is shared with other services	13/104 (12.5%)	2/58 (3.4%)^a^	4/30 (13.3%)^b^	7/16 (43.7%)
Only one ultrasound machine is available in the pulmonology service	34/104 (32.6%)	14/58 (2.1%)	14/30 (46.6%)	6/16 (37.5%)
No ultrasound scanner is available in the pulmonology service	6/104 (5.7%)	2/58 (3.4%)	2/30 (6.6%)	1/16 (6.25%)
Probe availability				
A linear, convex, and sector probe is available	37/104 (35.5%)	23/58 (39.6%)	9/30 (30%)	5/16 (31.2%)
A linear and convex probe is available	43/104 (41.3%)	26/58 (44.8%)	11/30 (36.6%)	6/16 (37.5%)
Only one probe is available	21/104 (20.1%)	9/58 (15.5%)	8/30 (26.6%)	4/16 (25%)
Linear	3/21	1/9	–	2/4
Convex	13/21	4/9	7/8	2/4
Microconvex	2/21	2/9	–	–
Sectorial	3/21	2/9	1/8	–
New applications				
Elastography is available	14/104 (13.4%)	9/58 (15.5%)	3/30 (10%)	2/16 (12.5%)
Elastography type				
Strain	6/104 (5.7%)	4/58 (6.8%)	1/30 (3.3%)	1/16 (6.2%)
SWE	0/104 (0%)	0/58 (0%)	0/30 (0%)	0/16 (0%)
Both	8/104 (7.6%)	5/58 (6.89%)	2/30 (6.6%)	1/16 (6.2%)
Elastography probe				
Linear	2/104 (1.9%)	1/58 (1.7%)	0/30 (0%)	1/16 (6.2%)
Convex	5/104 (4.8)	3/58 (5.1%)	2/30 (6.6%)	0/16 (0%)
Both	7/104 (6.7%)	5/58 (8.6%)	1/30 (3.6%)	1/16 (6.2%)
EBUS elastography is available	13/104 (12.5%)	11/58 (18.9%)	1/30 (3.3%)	1/16 (6.25%)
Dedicated LUS staff				
LUS is performed only in the bronchopleural procedures area	30/104 (28.8%)	18/58 (31.0%)	11/30 (36.6%)^b^	1/16 (6.25%)
LUS is performed in the bronchopleural procedures area and in the hospital ward on a routine basis	59/104 (56.7%)	32/58 (55.1%)	17/30 (56.6%)	10/16 (62.5%)
A person specialised in performing LUS is available	6/104 (5.7%)	4/58 (6.8%)	0/30 (0%)	2/16 (12.5%)

Data expressed as absolute frequencies (%)

*SWE* shear wave, *LUS* lung ultrasound, *EBUS* endobronchial ultrasound

^a^*p* < 0.05 high-complexity compared to low-complexity centres

^b^*p* < 0.05 intermediate-complexity compared to low-complexity centres

**Table 2:** Factors related to the portfolio of ultrasound services available

**SERVICES PORTFOLIO**	Total *N* = 104	High-complexity centre *N* = 58	Intermediate-complexity centre *N* = 30	Low-complexity centre *N* = 16
LUS is routinely used to diagnose and manage **PLEURAL EFFUSION**	95/104 (91.3%)	55/58 (94.8%)	27/30 (89.9%)	13/16 (81.2%)
LUS is routinely used to diagnose and/or follow-up **INTERSTITIAL PULMONARY DISEASES**	16/104 (15.3%)	10/58 (17.2%)	1/30 (3.3%)^b^	5/16 (31.2%)
LUS is routinely used to rule out **IATROGENIC PNEUMOTORAX** after bronchopleural procedures	53/104 (50.9%)	36/58 (62.0%)	10/30 (33.3%)	7/16 (43.7%)
LUS is routinely used for the diagnosis and/or **follow-up of PNEUMONIA**	32/104 (30.7%)	19/58 (32.7%)	7/30 (23.3%)	6/16 (37.5)
LUS is routinely used to guide **PULMONARY RECRUITMENT MANEUVERS**	6/104 (5.9%)	5/58 (8.6%)	0 (0%)	1/16 (6.25%)
LUS is routinely used to resolve** DIFFERENTIAL DIAGNOSES** between different pulmonary pathologies	54/104 (51.9%)	32/58 (55.1%)	15/30 (49.9%)	7/16 (43.7%)
LUS is routinely used to assess **VOCAL CORD DYSFUNCTION**	4/104 (3.8%)	2/58 (3.4%)	1/30 (3.3%)	1/16 (6.25%)
LUS is routinely used to diagnose and follow-up patients with **COVID-19**	29/104 (27.8%)	18/58 (31.0%)	6/30 (20%)	5/16 (31.2%)
**ECOSCOPY** is used for LVEF estimation, to measure cavities, for pericardial assessment, and to calculate the TAPSE	25/104 (24.0%)	15/58 (25.8%)	6/30 (20%)	4/16 (25%)
**LL ultrasound is routinely performed** when thromboembolic disease is suspected	28/104 (26.9%)	12/58 (20.6%)^a^	7/30 (23.3%)^b^	9/16 (56.2%)
**ELASTOGRAPHY is used to assess masses**	8/104 (7.6%)	7/58 (12.1%)	0 (0%)	1/16 (6.2%)
**ELASTOGRAPHY is used to assess pleural effusions**	6/104 (5.7%)	4/58 (6.8%)	1/30 (3.3%)	1/16 (6.2%)

*LL* lower limb, *COVID-19* coronavirus SARS-CoV2, *LVEF* left ventricular ejection fraction, *TAPSE* tricuspid annular plane systolic excursión

^a^*p* < 0.05 high-complexity compared to low-complexity centres

^b^*p* < 0.05 intermediate-complexity compared to low-complexity centres

**Table 3:** Factors related to teaching, training, and research in ultrasound

Teaching, training, and research		Total *N* = 104	High-complexity centre *N* = 58	Intermediate-complexity centre *N* = 30	Low-complexity centre *N* = 16
Accredited courses in pleural pathology are taught		21/104 (20.1%)	16/58 (27.5%)	4/30 (13.3%)	1/16 (6.2%)
Undergraduate teaching related to LUS is provided		36/104 (34.6%)	24/58 (41.3%)	9/30 (30%)	3/16 (18.7%)
Postgraduate teaching related to thoracic ultrasound is provided		32/104 (30.7%)	21/58 (36.2%)	9/30 (30%)	2/16 (12.5%)
Researchers working on their own thoracic ultrasound projects are present		18/104 (17.3%)	14/58 (24.1%)	3/30 (10%)	1/16 (6.2%)
There is participation in multicentre projects related to thoracic ultrasound		18/104 (17.3%)	15/58 (25.8%)^a^	3/30 (10%)	0/16 (0%)

^a^*p* < 0.05 high-complexity compared to low-complexity centres

### Statistical analysis

Qualitative variables were expressed as absolute frequencies and percentages. Comparisons between study groups were made according to the level of care provided at the hospital centre and the association between qualitative variables was evaluated with Chi-squared tests (*χ*^2^). The outcomes from all the statistical comparisons were considered significant when the probability of error was less than 5%. The data processing and analysis was carried out using SPSS software (v21, IBM Corp., Armonk, NY, USA).

### Ethical factors

Due to the nature of this study, neither informed consent nor approval by the Research Ethics Committee was required. The collection, treatment, and conservation of the data was carried out anonymously in accordance with the current provisions of the General Data Protection Regulation (EU Regulation 2016-679 of the European Parliament and Council, of April 27, 2016) and Spanish regulations on the protection of personal data.

## Results

### Sample description

A total of 155 health professionals belonging to 109 hospital centres responded to the survey. The results from three centres were excluded, because they were not located in the national territory and two other centres were excluded, because they were privately managed. Thus, data from 104 publicly managed hospitals were analysed in this work, of which 58 (55.8%) were highly complex centres, 30 (28.8%) were of intermediate complexity, and 16 (15.4%) were low-complexity centres.

### Results related to organisation and infrastructure

**Fig. 1:**
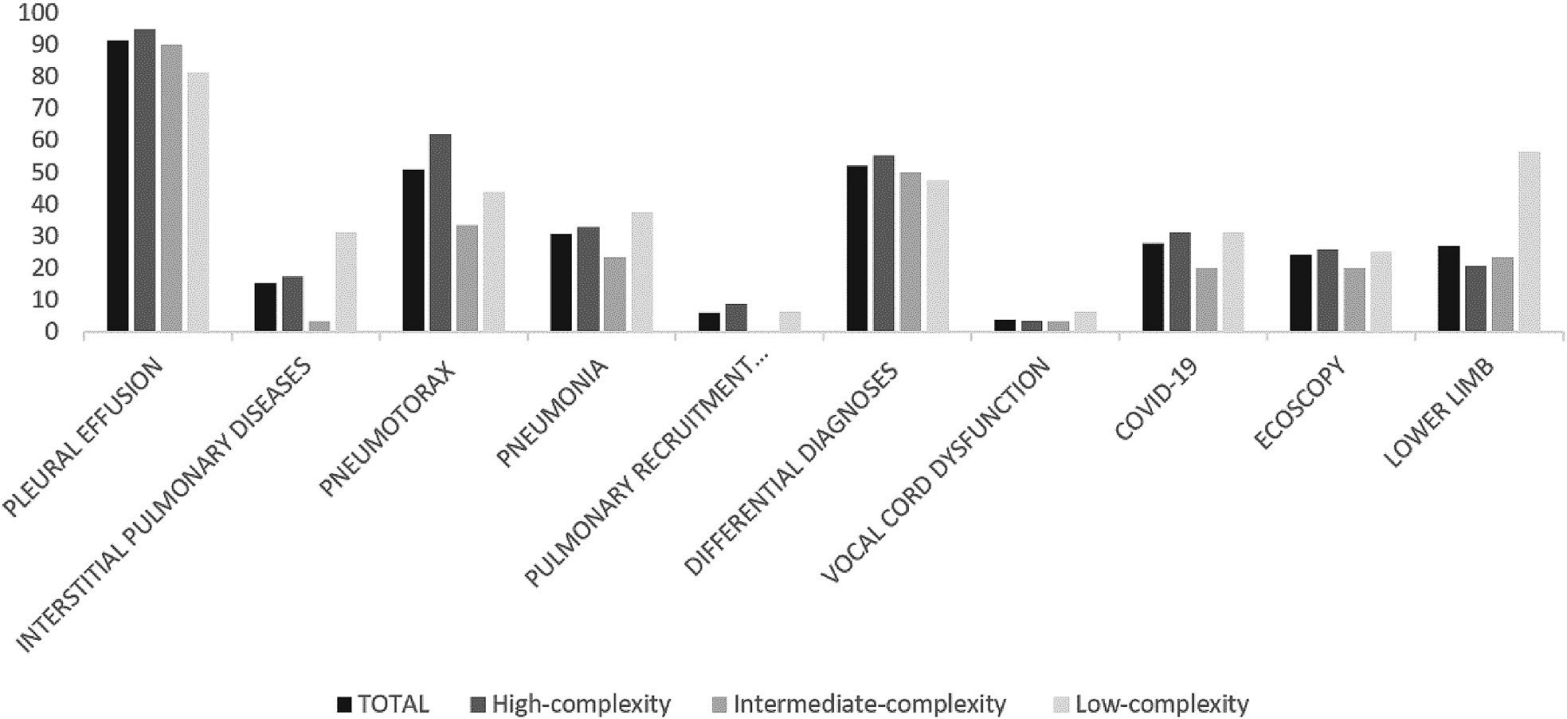
Factors related to the portfolio of ultrasound services available

We found significant differences in the availability of ultrasound machines depending on the complexity of the care provided at the centre. The pneumology service in high-complexity centres more often had at least 3 ultrasound scanners compared to low-complexity centres (37.9% vs. 0%; *p* = 0.008), with the latter being more likely to share an ultrasound system with other services (43.7% vs. 13.3% vs. 3.4%, respectively; *p* = 0.0001). We found no differences between the different levels of care in terms of the availability of different types of transducers; 35.5% of the centres surveyed had 3 types of transducer (convex, linear, and sector), 41.3% had linear and convex transducers, and 20% only had one type, usually a convex transducer (66.6%). LUS was routinely used (in 56.7% of cases) both in the bronchopleural procedures area and in the hospital ward, with no differences between the centre types. However, high- and intermediate-complexity care centres more often performed LUS exclusively in the bronchopleural procedures area compared to low-complexity centres (31% vs. 6.25%, *p* = 0.09 and 36.6% vs. 6.25%, *p* = 0.04, respectively). New ultrasonography techniques such as elastography were available in 13.4% of the centres, regardless of the level of care provided (Table 1, Fig. 1).

### Results related to the ultrasound service portfolio

LUS was most often used to evaluate pleural effusion (91.3%), to determine a differential diagnosis between pulmonary pathologies (51.9%), and to rule out iatrogenic pneumothorax after completing a broncho-pleural procedure (50.9%). The least common uses for LUS were for the evaluation of vocal cord dysfunction (3.8%) and the use of elastography to study masses (7.6%) and pleural effusions (5.7%). We only found significant differences for this item regarding the use of LUS for the diagnosis and/or follow-up of interstitial lung diseases (ILD) in low- vs. medium-complexity centres (31.2% vs. 3.3%, *p* = 0.02), and when performing lower limb (LL) ultrasound for suspected thromboembolic disease, which more often presented at low-complexity centres compared to intermediate (56.2% vs. 23.3%, *p* = 0.05) or high-complexity centres (56.2% vs. 20.6%, *p* = 0.01) (Table 2).

### Results related to teaching, training, and research

Overall, 34.6% and 30.7% of the centres taught undergraduate and postgraduate courses related to LUS and physicians in up to 17.3% of the centres were undertaking research projects related to LUS. In this sense, we only found significant differences in the number of multicentre research projects related to TE carried out at high-complexity centres vs. low-complexity centres (25.8% vs. 0%, *p* = 0.05).

## Discussion

This is the first study at a state level to assess the degree to which LUS is used by pulmonologist in Spain. Our data showed that LUS is widely used to study the pathologies for which it was first developed, but that training in new indications (for which never exceeded 65%) is required.

The assessment of pleural effusions showed the most consolidated use of LUS (91.3%), with the use of ultrasound improving the results obtained in this healthcare field compared to previous years. After the safety alert issued by the United Kingdom in 2008 [8] when a series of iatrogenic complications caused by the insertion of chest drains was identified, in 2010, the British Thoracic Society (BTS) recommended performing all pleural procedures with ultrasound guidance [9]. Nonetheless, a 2014 survey completed by 500 pulmonologists [10] found that up to 35% of LUSs used to guide these procedures were not yet performed by personnel specialised in pulmonology, and in a 2016 survey carried out among resident pulmonology and internal medicine physicians, only 44% said they routinely used LUS to perform thoracentesis [11].

In other recently introduced examinations, such as the diagnosis and follow-up of SARS-CoV2 pneumonia, LUS was used by 27.8% of the centres in this study, with no difference between the complexity of the centres. These results are lower than those obtained by the Academy of Thoracic Ultrasound [12] in Italy, who reported that 60.2% and 63.4% of their survey respondents used LUS for diagnosing and following up this pathology, respectively. However, this study was subject to selection bias, because the study cohort comprised clinicians with a special interest in LUS. Moreover, up to 14.6% of respondents started using LUS because of the COVID19 pandemic, thanks to the increased availability of equipment and training (through webinars, video tutorials, and local teaching programs) at the time. Thus, these two factors were key pillars in the development of this technique.

A 2018 study from Italy which attempted to clarify the main difficulties in establishing the regular use of LUS, found that a lack of ultrasound scanners (52%) and a deficit in training (22%) were the main barriers to more common use of the technique [13]. However, current accessibility to ultrasound scanners in our specific environment should favour the adequate development of this technique given that only 5.7% of the centres we surveyed did not have access to this equipment. Notwithstanding, this current work highlighted the need to improve the provision of global training in ultrasound, especially for new diagnostic techniques and for the evaluation of the heart, lower limbs, and vocal cords. Both in the Italian study and in the present one carried out in Spain, the most explored pathology by LUS was pleural effusion; however, in the Italian group, there is evidence of a higher degree of development of LUS for evaluation of pneumonia (55% vs. 30.7%), which could be justified by the inclusion, in the Italian survey, of personnel belonging not only to pulmonology but also to internal medicine or intensive care [13].

The continuous development of new and more complex ultrasound modalities, such as elastography [14, 15], means that more focussed training will be required to ensure adequate implementation of these techniques in clinical practice. Although the use of LUS appears in the postgraduate training program in pulmonology, the learning curve necessary to identify different pathologies is not well accounted for in this program. Proficiency in LUS is often developed at the bedside in clinical environments while supervised by a more experienced operator, or in specific training courses with simulation systems to acquire theoretical–practical knowledge [16]. LUS is not associated with any complications from the ultrasound technique, but clinical decisions made based on LUS mean that it is vital to professionally train operators to achieve high levels of diagnostic precision. Thus, to regulate and ensure training in LUS, the BTS published five levels of training in four main modalities, emergencies, basic, advanced, and expert [17], ranging from an initial degree as an observer to an expert level with permission to supervise and train other professionals. All pulmonologists in training must reach the basic level and can go on to complete the advanced levels, while the expert level would be assigned to people specifically specialised in ultrasound; in our sample this corresponded to only 5.7% of the population (Table 1).

Regarding organisational and infrastructural factors, with respect to low-complexity hospitals, LUS tended to be performed only in the bronchopleural procedures area in high and intermediate-complexity centres (6.15% vs. 31% and 36.6%, respectively). However, of note, a significantly wider range of techniques, such as the evaluation of DVT, were carried out in low-complexity centres (56.2% vs. 20.6% and 23.3%, respectively), perhaps because more complex centres are more subspecialised.

Only 20.1% of the surveyed centres taught accredited courses, with this figure being slightly higher than for high-complexity centres. Regarding research, there were very few institutional or multicentre projects being carried out in relation to ultrasound. Thus, we consider this as another objective for improvement, because such research translates into improvement of the quality of care, which is both a corporate and healthcare provision goal.

The limitations of this current study included the low percentage of professionals included that belonged to low-complexity centres, the lack of stratification by autonomous communities, which prevented us from performing an analysis of variability between territories, and that possible areas of development of LUS, such as consultations or critical units, have not been included. However, in our opinion, the analysed sample was sufficient to reflect how LUS is currently used in Spain and allowed us to establish recommendations for the better management of these processes. At the national level, other research groups have developed surveys to investigate the use of LUS in other prevalent pathologies, such as COPD [18], for the management of pleural effusion [7, 11] or in palliative care units [19]. We believe this type of analysis is required to develop new strategic lines of research through scientific societies, because ultrasound could be used to internally evaluate the quality of healthcare provision in different services, develop guidelines to improve the quality of care, promote the development of training, and to facilitate comparative studies between different hospitals.

## Conclusions

Ultrasound is a tool whose use has increased in recent years, not only in bronchopleural procedure units, but also in the context of conventional ward hospitalisation, intermediate care units, and consultations. In parallel, the availability of ultrasound machines in pneumology services has also increased as a result of the clear advantages of this technique, which is why it seems necessary to gradually implement ultrasound as the fifth pillar of the physical examination of every type of pulmonary pathology. Nonetheless, this implies that pulmonologists must complete training to acquire advanced knowledge in this technique.

## Data Availability

The data sets used and/or analysed during the current study are available from the corresponding author on reasonable request.

## Abbreviations

BTS: British Thoracic Society
COVID-19: Coronavirus disease
DVT: Deep vein thrombosis
EBUS: Endobronchial ultrasound
EU: Europe
ILD: Interstitial lung disease
LVEF: Left ventricular ejection fraction
LL: Lower limb
LUS: Lung ultrasound
MIRs: Medical intern residents
SEPAR: Spanish Society of Pulmonology and Thoracic Surgery
SWE: Shear wave
TAPSE: Tricuspid annular plane systolic excursion
